# Effects of Chronic Hypoxia on the Immune Status of Pikeperch (*Sander lucioperca* Linnaeus, 1758)

**DOI:** 10.3390/biology10070649

**Published:** 2021-07-12

**Authors:** Nadine Schäfer, Jan Matoušek, Alexander Rebl, Vlastimil Stejskal, Ronald M. Brunner, Tom Goldammer, Marieke Verleih, Tomáš Korytář

**Affiliations:** 1 Fish Genetics Unit, Institute of Genome Biology, Leibniz Institute for Farm Animal Biology (FBN), 18196 Dummerstorf, Germany; schaefer@fbn-dummerstorf.de (N.S.); rebl@fbn-dummerstorf.de (A.R.); brunner@fbn-dummerstorf.de (R.M.B.); tom.goldammer@uni-rostock.de (T.G.); 2 Institute of Aquaculture and Protection of Waters (IAPW), Faculty of Fisheries and Protection of Waters, University of South Bohemia, 370 05 České Budějovice, Czech Republic; matouj03@frov.jcu.cz (J.M.); stejskal@frov.jcu.cz (V.S.); 3 Molecular Biology and Fish Genetics, Faculty of Agriculture and Environmental Sciences, University of Rostock, 18059 Rostock, Germany; 4 Institute of Parasitology, Biology Centre CAS, 370 05 České Budějovice, Czech Republic

**Keywords:** pikeperch, hypoxia, intraperitoneal stimulation, immune response, stress response, *Aeromonas hydrophila*

## Abstract

**Simple Summary:**

Inadequate oxygen saturation, or hypoxia, belongs to one of the critical stress factors in intensive aquaculture. Exposure of fish to low oxygen levels over prolonged periods substantially affects their well-being and immune competence, resulting in increased disease susceptibility and consequent economic losses. In this interdisciplinary research, we aimed to provide a deeper understanding of the effect of chronic low oxygen saturation on pikeperch farmed in recirculating aquaculture systems. The obtained data offer unprecedented insights into the changes in the immunocompetence of studied fish and suggest high robustness of this new aquaculture species to the stress factors of intensive aquaculture.

**Abstract:**

Inadequate oxygen saturation can induce stress responses in fish and further affect their immunity. Pikeperch, recently introduced in intensive aquaculture, is suggested to be reared at nearly 100% DO (dissolved oxygen), yet this recommendation can be compromised by several factors including the water temperature, stocking densities or low circulation. Herein, we aimed to investigate the effect of low oxygen saturation of 40% DO (±3.2 mg/L) over 28 days on pikeperch farmed in recirculating aquaculture systems. The obtained data suggest that—although the standard blood and health parameters did not reveal any significant differences at any timepoint—the flow cytometric analysis identified a slightly decreased proportion of lymphocytes in the HK (head kidney) of fish exposed to hypoxia. This has been complemented by marginally downregulated expression of investigated immune and stress genes in HK and liver (including *FTH1*, *HIF1A* and *NR3C1*). Additionally, in the model of acute peritoneal inflammation induced with inactivated *Aeromonas hydrophila*, we observed a striking dichotomy in the sensitivity to the low DO between innate and adaptive immunity. Thus, while the mobilization of myeloid cells from HK to blood, spleen and peritoneal cavity, underlined by changes in the expression of key proinflammatory cytokines (including *MPO*, *IL1B* and *TNF*) was not influenced by the low DO, hypoxia impaired the influx of lymphocytes to the peritoneal niche in the later phases of the immune reaction. Taken together, our data suggest high robustness of pikeperch towards the low oxygen saturation and further encourage its introduction to the intensive aquaculture systems.

## 1. Introduction

Low dissolved oxygen (DO) levels induce primary, secondary and tertiary stress responses in fish [1]. Optimal oxygen saturation is a vital parameter for animals and, therefore, tightly controlled in intensive aquaculture facilities. Hypoxia is defined as depletion of oxygen concentration, which can substantially affect the fish’s well-being and immune status, resulting in increased susceptibility to stressors and reduced resistance to pathogens [2]. Bregnballe (2015) defined oxygen saturations below 40% (equivalent to ~3–4 mg/L at 20–25 °C) as inadequate for aquaculture facilities in general [3]. The required concentration of dissolved oxygen is dependent on the fish species and the corresponding water parameters. Furthermore, the animal-specific sensitivity to low oxygen saturation and the duration and the intensity of hypoxic conditions influence the outcome of the triggered response [4,5]. There are several reasons for low DO levels in aquaculture systems, including inadequate water circulation [6], high water temperatures [7] and high stocking densities [8]. Percids seem to be relatively tolerant to low DO levels [9,10]. For instance, feeding rate of yellow perch (*Perca flavescens*) consumption is negatively regulated only with oxygen levels of 3.5 mg/L and lower [11]. The growth of walleye (*Sander vitreus*) and yellow perch is affected by oxygen levels only below 2 mg/L [12,13,14]. Nevertheless, levels below 5 mg/L DO can induce significant stress responses in Eurasian perch (*Perca fluviatilis*) [15].

The pikeperch (*Sander lucioperca*) is native to fresh and brackish waters of the northern hemisphere [16], and due to its high-quality flesh and high market acceptance, it became a significant food fish for regional aquaculture in Europe [17]. Within its natural habitat, the DO levels range from 5.5 to 12.9 mg/L [18,19]. Thus far, nearly 100% DO (equivalent to ~7–9 mg/L DO at 20–25 °C) has been suggested as the optimal oxygen saturation for pikeperch aquaculture [20,21,22]. However, the lower limit of the pikeperch’s tolerance to low DO levels under intensive farming, and its impact on physiology and immunity remains vague. Previously, Stejskal et al. (2012) observed that a 50–60% oxygen saturation (equivalent to ~4–6 mg/L DO at 20–25 °C) correlates with lower feed intake and a reduced growth rate in intensive pikeperch farming [23], while in independent study similar oxygen saturation for 36 days led to a significant increase in the complement activity of the sera [24]. Nevertheless, a more detailed understanding of the effect of chronic low oxygen saturation on immune system of pikeperch is absent. In this study, we aimed to fill this gap and elucidate how low oxygen saturation of 40% (±3.2 mg/L DO) for 28 days influences the standard health and immune system parameters and expression of selected genes involved in the regulation of immune and stress responses. Furthermore, to gain further insights into the capacity of the immune system to induce inflammatory responses under low DO, we employed a previously established model of acute peritonitis with *Aeromonas hydrophila*, Gram-negative bacteria associated with mortality of fish kept under adverse environmental conditions [25,26].

## 2. Materials and Methods

### 2.1. Fish Rearing and Experimental Design

**Figure 1:**
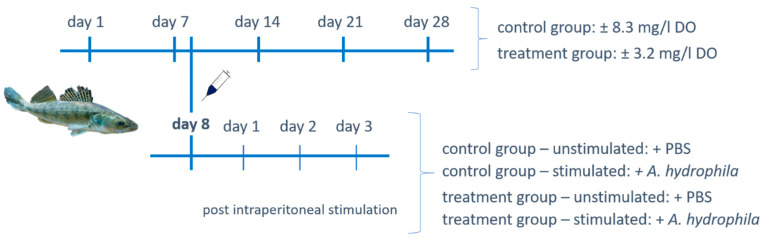
Outline of the experimental design. The 98 adult pikeperch were kept either under normoxic water conditions (±8.3 mg/L dissolved oxygen (DO) level) or low DO saturation (±3.2 mg/L DO level) for up to 28 days. Peripheral blood, head kidney (HK) and liver tissues were sampled from five fish of both groups. Additionally, at day 8 of the experiment, 48 fish were intraperitoneally injected with either 1 × 10^7^ inactivated *Aeromonas hydrophila* cells in 100 µL sterile phosphate-buffered saline solution (PBS) or exclusively 100 µL PBS. At three following days, peritoneal leukocytes, peripheral blood, HK and spleen were collected from four fish per group.

Ninety-eight juvenile pikeperch (obtained from Anapartners, s.r.o., Prague, Czech Republic), with an average length of 29.51 ± 0.47 cm and body weight of 219.39 ± 9.79 g, were reared in a recirculating aquaculture system (RAS) at the Institute of Aquaculture and Protection of Waters (IAPW; České Budějovice, Czech Republic) from June to August 2018. Fish were randomly assigned in six identical black plastic 300 L tanks and acclimated for two weeks at 23.1 ± 1.0 °C with 12:12 h day/night light period and a light intensity of 20–35 Lx. Water was pre-treated by drum and moving bed filters in complement with UV disinfection and aeration. In all tanks, the inflow-outflow rate was 6 L/min, generated through mixing towers, and in-tank oxygen saturation was monitored online using the controller HACH SC 1000 (HACH Lange, Düsseldorf, Germany). Feeding was performed with a commercial extruded diet (EFICO Sigma 970, 3 mm, BioMar A/S, Brande, Denmark) by automatic feeders (EHEIM Twins, Deizisau, Germany; 6 meals per day) and one hand feeding. Fish were fed ad libitum. The experimental design is illustrated in Figure 1. The experiment was performed in triplicate tanks for up to 28 days, with ±3.2 mg/L DO levels (40% oxygen saturation) for “low DO group” (total n = 49; 12–24 per tank) and ±8.3 mg/L DO levels (>95% oxygen saturation) for the control group (total n = 49; 12–24 per tank). Low DO conditions were established by additional nitrogen administration according to the oxygen depletion system generated by Pichavant et al. (2000) [27], including individual modifications. Water quality parameters (NH_3_, NH^4+^, NO^2−^ and NO^3−^) were monitored throughout the experiment in the two-day interval using commercial kits (LCK 304, LCK 341, LCK 339 and HACH Lange, Düsseldorf, Germany) and spectrophotometric analyses (DR 3900 and HACH Lange, Düsseldorf, Germany). The concentration of ammonia, nitrite, and nitrate was 0.24 ± 0.11, 0.21 ± 0.13 and 4.54 ± 4.18, respectively. Temperature and pH were monitored daily with HACH HQ40 multimeter and reached 23.5 ± 0.7 °C and 7.38 ± 0.25, respectively.

Before sampling, fish were anesthetized with 30 µL/L clove oil and stunned in compliance with the terms of the Czech legislation (Section 29 of Act No.246/1992 Coll., on Protection of animals against cruelty, as amended by Act No. 77/2004 Coll.). All animal experiments have been approved by the Ministry of Education, approval ID: MSMT-18301/2018-2.

On days one, seven, fourteen, twenty-one and twenty-eight of the treatment, we sampled peripheral blood, head kidney (HK) and liver of five fish per control and low DO group. Additionally, to elucidate the impact of hypoxia on acute inflammation, we performed peritoneal stimulation described below. Upon induction of peritoneal inflammation, peripheral blood, HK, spleen and the peritoneal leukocytes were sampled at day one, two and three post-stimulation at days nine, ten and eleven of the hypoxia experiment. Peripheral blood was collected from the caudal vein with heparinized (Sigma-Aldrich, Taufkirchen, Germany) syringes. Parts of the HK, liver, and spleen were snap-frozen in liquid nitrogen and stored at −80 °C until RNA isolation. The peritoneal leukocytes were obtained from peritoneal lavage as described previously [28].

### 2.2. Intraperitoneal Stimulation with Aeromonas hydrophila

To evaluate the impact of low DO on the acute inflammatory response, fish were stimulated with 1.5% paraformaldehyde (PFA) inactivated *A. hydrophila*. To this end, 48 pikeperch (24 each per control and low DO group, 208.85 ± 6.58 g) were intraperitoneally injected at day eight of the experiment (Figure 1) with either a total of 1 × 10^7^ *A. hydrophila* in 100 μL sterile phosphate-buffered saline solution (PBS) or exclusively with 100 µL PBS.

### 2.3. Cell Isolation

The blood samples were diluted 1:150 in DMEM (Dulbecco’s modified eagle medium; Gibco/Life Technologies, Carlsbad, CA, USA) on ice. The remaining HK and spleen were homogenized by cell strainer (100 μm; Corning Inc., Corning, NY, USA) and resuspended in 4 mL DMEM (Gibco) on ice. Collected cells were further 1:1 diluted in DMEM (Gibco) on ice, layered onto an isotonic Percoll^TM^ (Ge Healthcare, Uppsala, Sweden) gradient (34% plus 51%; r = 1.075 g/mL) on ice and centrifuged at 800× *g* for 15 min at 8 °C. We collected the HK leukocytes at the interphase of the different Percoll™ concentrations, washed the cell suspension and, finally, resuspended it in 500 μL DMEM (Gibco) on ice. Cells were further 1:2 diluted in DMEM (Gibco) on ice and applied for flow cytometry.

### 2.4. Flow Cytometry

To investigate the cell composition of peripheral blood, HK and spleen samples we applied flow cytometry analysis using the BD FACSCanto™ II system (BD Biosciences, Prague, Czech Republic) in medium flow rate (1 µL/s). Briefly, the leukocytes from HK and spleen were diluted to a concentration 1 × 10^6^/mL and 100 µL of the cell suspension was used for the measurement. Furthermore, 2 µL of whole blood were diluted in 200 µL of DMEM containing the DIOC6 dye and recorded for 20 s at a constant flow of 1 µL/second. To determine the total number of peritoneal leukocytes, we employed the flow cytometry protocol described previously [28]. The cell morphology was evaluated using the FSC-SSC parameters, with lymphocytes being FSC^lo^-SSC^lo^ and myeloid cells being defined as FCS^hi^-SSC^hi^.

### 2.5. Health Parameters

We measured traditional health indicators, including total length, weight, spleno- (SSI) and hepato-somatic indices (HSI), as well as concentrations of glucose, lactate and plasma cortisol levels. The SSI was calculated by the formula: spleen weight (g)/body weight (g) × 100; the HSI was calculated by the formula: liver weight (g)/body weight (g) × 100. Whole blood glucose and lactate levels were analyzed using the Accutrend^®^ Plus device (Cobas; Roche Diagnostics, Mannheim, Germany). Plasma cortisol levels were measured by the Cortisol ELISA assay (DRG Instruments, Marburg, Germany) according to the manufacturer’s instructions.

### 2.6. Gene Selection and Primer Design

To evaluate the transcriptional response to low DO saturation and the capacity of fish to induce inflammatory responses under stress, we established a screening panel with 15 genes involved in stress and immune response (listed in Table 1).

The genes, elongation factor 1 alpha (*EEF1A1*), ribosomal protein L32 (*RPL32*) and ribosomal protein S5 (*RPS5*) were applied as reference according to Swirplies et al. (2019) [29]. For the candidate genes interleukin 8 (*CXCL8*), hypoxia-inducible factor 1 subunit alpha (*HIF1A*), heme oxygenase 1 (*HMOX1*), heat shock transcription factor 1 (*HSF1*), heat shock transcription factor 2 (*HSF2*), heat shock protein 90 alpha family class A member 1 (*HSP90AA1*), interleukin 1 beta (*IL1B*), nuclear receptor subfamily 3 group c member 1 (*NR3C1*) and tumor necrosis factor (*TNF*) pikeperch-specific oligonucleotide sequences were already available from our former studies [29,30]. Using our recently published pikeperch genome (RefSeq NCBI: GCA_008315115.1) [31], we identified the remaining orthologs for *S. lucioperca*. The Pyrosequencing Assay Design software (version 1.0.6; Biotage, Uppsala, Sweden) was applied to derive optimal oligonucleotide primers. We purchased all primers from Sigma-Aldrich, Taufkirchen, Germany) and validated them by sequencing their PCR products (Applied Biosystems 3130 Genetic Analyzer; Life Technologies, Carlsbad, CA, USA).

### 2.7. RNA/cDNA Preparation

Total RNA was extracted from collected samples by homogenizing tissues (HK, liver and spleen) separately within 1 mL TRIzol Reagent (Invitrogen/Thermo Fisher Scientific, Karlsruhe, Germany), as stated in the manufacturer’s instructions. Subsequently, we purified all samples with the RNeasy Mini Kit (Qiagen, Hilden, Germany), including DNAse treatment. For isolated HK leukocytes, 3.5 µL of 2-mercaptoethanol (Sigma-Aldrich, Taufkirchen, Germany) was added, and samples were purified with the ISOLATE II RNA Mini Kit (Bioline/Meridian Bioscience, London, UK). Applying gel electrophoresis and spectrophotometry analysis in repeated measurements (ND 1000; NanoDrop Technologies/Thermo Fisher Scientific, Waltham, MA, USA), the quantity and quality of the isolated nucleic acids were determined. Collected RNA was stored at −80 °C until further application.

Synthesis of cDNA was performed from 1.0–1.5 µg of total RNA using the SuperScript II Reverse Transcriptase Kit (Thermo Fisher Scientific, Karlsruhe, Germany) according to the manufacturer’s protocol, and cDNA was stored at −20 °C.

### 2.8. Real-TIME Quantitative PCR (rt-qPCR)

The gene expression during low DO levels was evaluated by real-time quantitative PCR (rt-qPCR). Therefore, we implemented the SensiFAST™ SYBR No-ROX Kit (Bioline, Luckenwalde, Germany) and the LightCycler96 system (Roche, Basel, Switzerland). PCR conditions were as follows: the initial denaturation step (95 °C, 5 min) was followed by 40 cycles of denaturation (95 °C, 15 s), annealing (60 °C, 10 s), elongation (72 °C, 20 s) and a fluorescence measurement step for 10 s (75 °C). For the copy-number calculation by linear regression analysis (R^2^ > 0.999), standard curves based on Cq values of tenfold dilutions of the generated fragments (1 × 10^3^–1 × 10^8^ copies) were generated. Cq values > 35 were considered as not detectable. For confirmation of the quality of all PCR products, we conducted melting curve analysis and gel electrophoresis.

Three reference genes (*EEF1A1*, *RPL32* and *RPS5*) for *S. lucioperca* [29] were evaluated for each sample and applied for data normalization.

### 2.9. Statistics

Rt-qPCR data were evaluated with the LightCycler 96 software v. 4.0.1. Flow cytometry data were analyzed by using the BD FACSDiva software FlowJow10. Statistically significant differences in blood parameters, gene expression and cell composition between control and low DO group were determined per day using the multiple t-test (Holm–Šídák corrected, α = 0.05). For the additional stimulation experiment data, statistical significances were calculated with one-way ANOVA followed by Tukey’s multiple comparison test (*p* < 0.05).

## 3. Results

### 3.1. Reduced Oxygen Saturation Induces Marginal Changes in the Process of Adaptation

#### 3.1.1. Blood and Health Parameters of Challenged Pikeperch

To evaluate the physiological responses of the fish challenged with the low DO levels, we recorded standard health parameters including blood glucose, lactate and plasma cortisol levels, as well as SSI and LSI throughout the experiment. Notably, these parameters did not reveal any significant differences between the “control” (±8.3 mg/L DO) and “low DO group” (±3.2 mg/L DO) at any time point of the experiment (Supplementary Table S1).

#### 3.1.2. Composition of HK and Peripheral Blood upon Low DO Exposure

**Figure 2:**
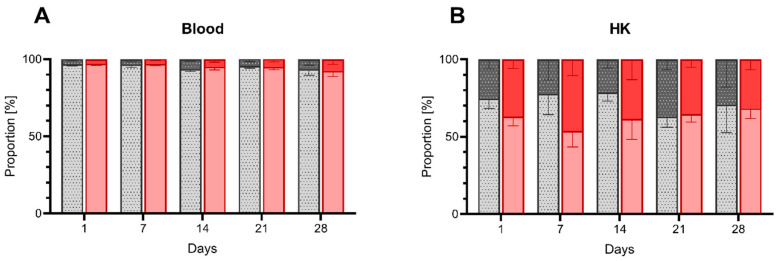
Proportion of myeloid and lymphoid cells within blood and HK of pikeperch challenged with low DO conditions. Proportion (%) of lymphoid (bright color) and myeloid cells (dark color) in collected blood (**A**) and head kidney (HK; (**B**)) samples of pikeperch. HK was additionally purified by Percoll-gradient. Columns represent mean of five individual samples (+SEM) from control (grey/patterned) and hypoxia group (red) after one, seven, fourteen, twenty-one and twenty-eight days of the experiments. Statistical significance per cell type per each day was determined using the multiple t-test (Holm–Šídák corrected), with alpha = 0.05.

We hypothesized that inadequate oxygen saturation would affect the immune status of the fish, reflected by a change in the proportion of immune cells. Therefore, we employed flow cytometry to analyze the ratio between myeloid and lymphoid cells in HK and peripheral blood. (Figure 2).

Throughout the experiment, the average cell composition of the peripheral blood leukocytes remained almost unchanged between the control and the low DO group, with a proportion of approximately 95% lymphocytes to 5% myeloid cells (Figure 2A). Conversely, the composition of HK exhibited notable differences between both groups. Thus, while the ratio between myeloid cells and lymphocytes in the control group underwent only mild fluctuations ranging from 63% to 79% of lymphocytes and 21% to 37% of myeloid cells, we observed more substantial changes in the group exposed to low DO. Particularly at the early time points (day one, seven and fourteen), we witnessed an increase in the proportion of myeloid cells, reaching to 46%, reflected by a 1.4× decrease in lymphocyte proportion (Figure 2B).

#### 3.1.3. Gene Expression Analysis in HK and Liver of Challenged Pikeperch

**Figure 3:**
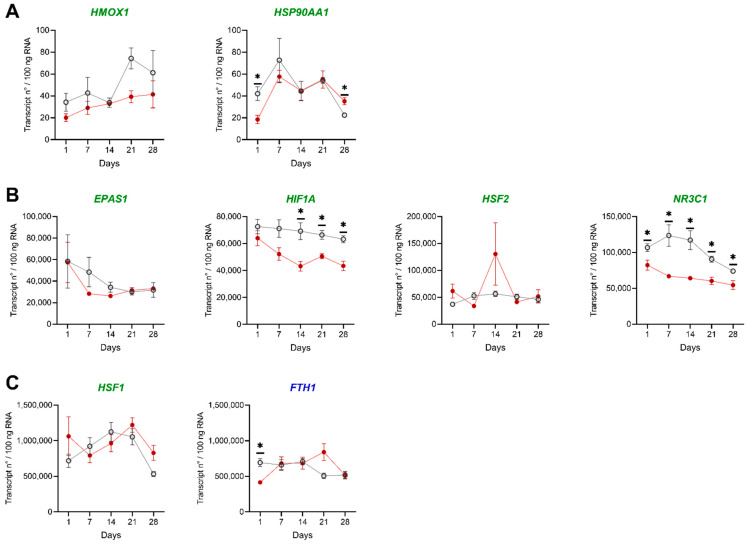
Stress and immune marker expression in HK of pikeperch exposed to low oxygen saturation. Gene expression of candidate genes in collected head kidney (HK) samples of pikeperch. Normoxic (grey/empty circles) and low DO (red/filled circles) groups after one, seven, fourteen, twenty-one and twenty-eight days of the experiment. Genes involved in stress (green) or immune response (blue) were either lowly (**A**), moderately (**B**) or highly (**C**) expressed. Data points represent the mean of five individual samples (+SEM), calculated per 100 ng of total RNA. Statistical significance was determined between groups for each day using the multiple t-test (Holm–Šídák corrected), with alpha = 0.05; * < 0.05.

**Figure 4:**
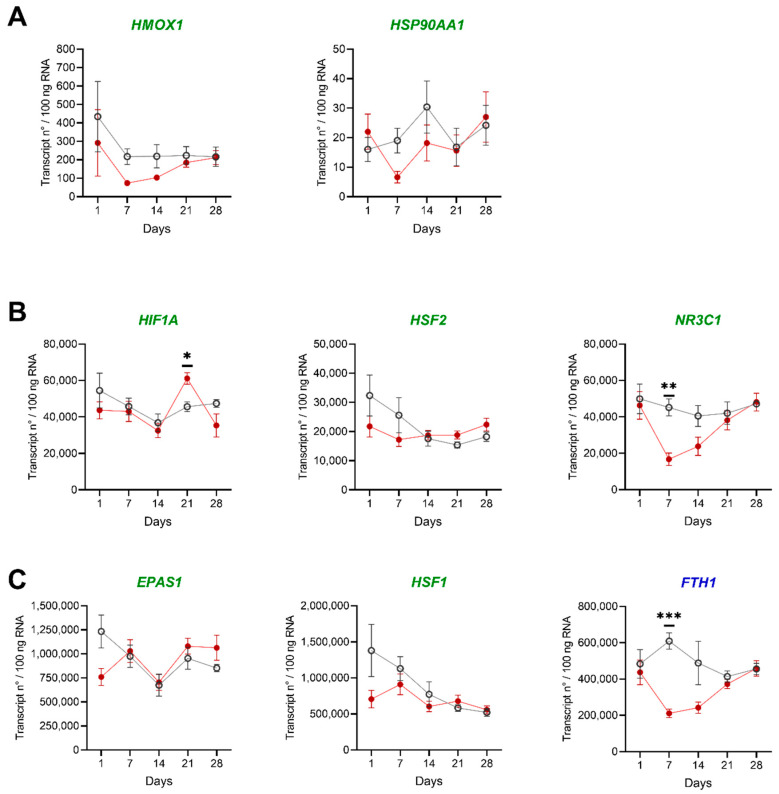
Stress and immune marker genes in liver of pikeperch challenged with low oxygen saturation. Gene expression of candidate genes in collected liver samples of pikeperch. Normoxic (grey/empty circles) and low DO (red/filled circles) group after one, seven, fourteen, twenty-one and twenty-eight days of the experiment. Genes involved in stress (green) or immune response (blue) are either lowly (**A**), moderately (**B**) or highly (**C**) expressed. Data points represent the mean of five individual samples (+SEM), calculated per 100 ng of total RNA. Statistical significance was determined between groups for each day using the multiple t-test (Holm–Šídák corrected), with alpha = 0.05; * < 0.05, ** < 0.01 and *** < 0.001.

To provide deeper insights into the molecular mechanisms underlying the changes induced by low DO levels, we evaluated the expression of eight selected genes involved in response to hypoxia. The transcript numbers ranged from approximately 2 × 10^1^ (*HSP90AA1*) to 2.5 × 10^6^ (*HSF2*) copies per 100 ng RNA in HK and liver of individual fish (Figure 3A–C and Figure 4A–C). We detected transcript numbers only below 2 × 10^3^ transcripts/100 ng RNA for the genes *HMOX1* and *HSP90AA1* in HK and liver (Figure 3A and Figure 4A). The highest copy numbers with 1.5 × 10^5^ transcripts/100 ng RNA and above were observed for the genes *EPAS1*, *FTH1* and *HSF1* in HK and liver (Figure 3C and Figure 4C).

In general, few genes were lower expressed under low DO levels in liver and HK than in the control group. Three of the analyzed genes (*EPAS1* in HK; *FTH1* and *NR3C1* in the liver) shared similar transcript patterns. Here, the expression levels of both groups are similar at the beginning and the end of the experiment, but at days seven and fourteen the low DO group showed lower transcript levels.

Four genes showed statistically different copy numbers between the control and the low DO group. For *HSP90AA1*, a change from significantly lower to significantly higher copy numbers during the treatment was observed in HK. *HIF1A* showed higher transcript levels during the low DO exclusively at day 2 in the liver, but lower copy numbers from day 14 till day 28 in HK. Another two genes (*FTH1* and *NR3C1*: HK and liver) showed lower transcript levels during the low DO challenge.

### 3.2. Induction of Peritoneal Inflammation under Low DO Levels

#### 3.2.1. SSI after Intraperitoneal Stimulation

**Figure 5:**
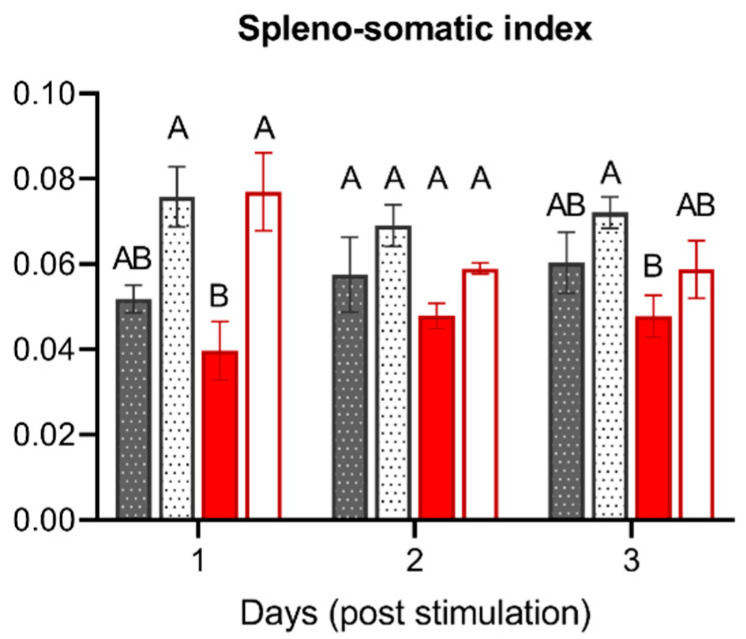
Spleno-somatic index of pikeperch during acute peritoneal inflammation. Spleno-somatic indices of pikeperch after one, two and three days post intraperitoneal stimulation with inactivated *Aeromonas hydrophila* cells. The graph shows differences between normoxic control groups, either unstimulated (dark grey/patterned) or stimulated (bright grey/patterned), and low DO groups, either unstimulated (dark red) or stimulated (bright red). Columns represent mean of four individual samples (+SEM). Statistical significance per each day was determined using the one-way ANOVA followed by Tukey´s multiple comparison test (*p* = 0.05); different letters (A, B) represent significant changes between different control and low DO groups.

To elucidate to which extent is the immune response of the host compromised by the reduced levels of DO, on day eight (Figure 1), we employed an adapted model of peritoneal inflammation established previously [28]. Upon the stimulation, we determined the SSI in all tested groups, the control group and low DO group, both either unstimulated (PBS as control) or stimulated with inactivated *A. hydrophila* (Figure 5).

The average spleno-somatic indices ranged from 0.040 to 0.077. The injection of inactivated bacteria resulted in an increase of the SSI in both the control and the low DO group at day one to three post-stimulation, with the most prominent and significant changes seen between unstimulated and stimulated fish of the low DO group at day one. Furthermore, we noticed a slight, albeit nonsignificant, decrease in the SSI in the PBS-injected fish in the low DO group compared to normoxia.

#### 3.2.2. Leukocyte Migration upon Intraperitoneal Stimulation

**Figure 6:**
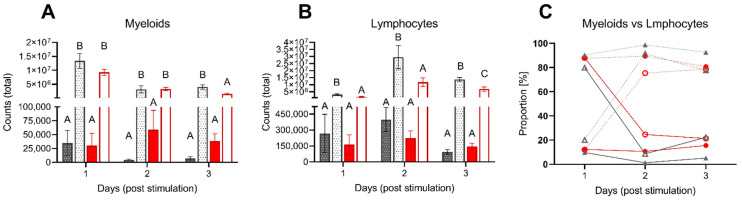
Kinetics of peritoneal leukocytes upon acute peritoneal inflammation. Total counts of myeloid cells (**A**), lymphocytes (**B**) and proportion of both cell types (**C**) after one, two and three days post intraperitoneal stimulation with inactivated *Aeromonas hydrophila* cells. Graphs A and B show differences between normoxic control groups, either unstimulated (dark grey/patterned) or stimulated (bright grey/patterned), and low DO groups, either unstimulated (dark red) or stimulated (bright red). Columns represent mean of four individual samples (+SEM). Statistical significance per each day was determined using the one-way ANOVA followed by Tukey’s multiple comparison test (*p* = 0.05); different letters (A–C) represent significant changes between different groups. Graph C shows differences between control groups, either unstimulated (grey, filled triangle) or stimulated (grey, open triangles), and the low DO groups, either unstimulated (red, filled circles) or stimulated (red, open circles). Dotted lines represent lymphocytes, filled lines represent myeloid cells.

**Figure 7:**
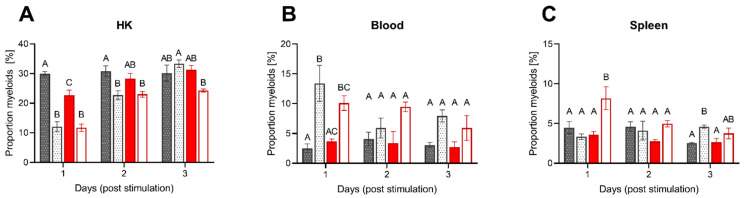
Proportion of myeloid cells within HK, peripheral blood and spleen of pikeperch upon intraperitoneal stimulation. Proportion (%) of myeloid cells in head kidney (HK; **A**), peripheral blood (**B**) and spleen (**C**) samples of pikeperch after one, two and three days post intraperitoneal stimulation with inactivated *Aeromonas hydrophila* cells. The graph shows differences between normoxic control groups, either unstimulated (dark grey/patterned) or stimulated (bright grey/patterned), and low DO groups, either unstimulated (dark red) or stimulated (bright red). Columns represent mean of four individual samples (+SEM). Statistical significance per each day was determined using the one-way ANOVA followed by Tukey´s multiple comparison test (*p* = 0.05); different letters (A–C) represent significant changes between different groups.

To further evaluate the impact of low DO on acute inflammation, we analyzed the cell composition in the peritoneal cavity, blood, spleen and head kidney upon intraperitoneal injection with *A. hydrophila* (Figure 6A–C and Figure 7A–C).

While the PBS-injected fish did not undergo any remarkable changes in the number of cells in the peritoneal cavity and retained their original composition with 80–90% of lymphocytes and 10–20% of myeloid cells throughout the experiment, the injection of inactivated *A. hydrophila* led to rapid recruitment of myeloid cells to the peritoneal cavity, resulting in a complete change in its profile (Figure 6A–C). As soon as one day after the injection, the myeloid cells comprised over 80–90% of all peritoneal leukocytes (Figure 6C), reaching the total number of 1.4 × 10^7^ and 9 × 10^6^ in control and DO low groups, respectively (Figure 6A). In the following 24 h, the peritoneal niche underwent the second change in its composition, and the growing number of lymphocytes substituted the peak of myeloid cells. Their total number reached approximately 3 × 10^7^ cells in the control group (Figure 6B), representing over 80% of all peritoneal leukocytes (Figure 6C). Notably, the recruitment of lymphocytes to the peritoneum of low DO fish was three times lower than in fish kept at normal oxygen saturation. On the third day, we observed a resolution of inflammation in both stimulated groups.

The recruitment of leukocytes to the peritoneal cavity was mirrored by the changes in the composition of blood and both systemic lymphoid organs. The changes were most prominent on the first two days when the stimulation with inactivated *A. hydrophila* decreased the proportion of myeloid cells in the HK from 30% observed in PBS injected controls to ~12% and ~23%, respectively (Figure 7A). In addition, we observed a significant decrease in the myeloid proportion of the low DO group (~23%) after the first day (without additional stimulation) compared to the control group (~30%) (Figure 7A). This decrease was complemented by the increased mobilization of myeloid cells to the peripheral blood, which increased from ~3% in PBS injected groups up to 13% in stimulated fish 24 h post-injection (Figure 7B). This has been further reflected by the increased ratio of myeloid cells in the spleen of low DO fish but not in the fish kept at standard oxygen saturation (Figure 7C). With the ensuing resolution of the inflammation in the peritoneal cavity, the proportion of myeloid cells in the blood decreased gradually to ~8%.

#### 3.2.3. Gene Profiling in HK and Spleen during Acute Inflammation

**Figure 8:**
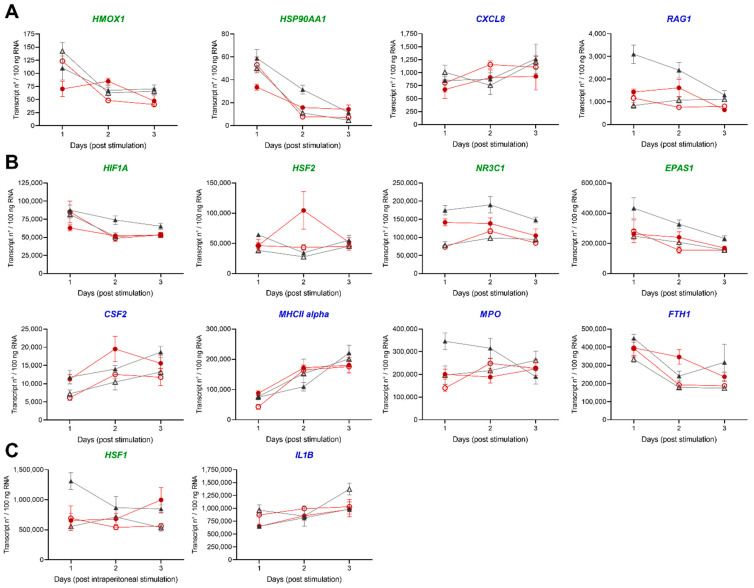
Expression of stress and immune markers in HK of pikeperch upon acute peritoneal inflammation. Expression of candidate genes in head kidney (HK) of pikeperch. Normoxic control group (grey), either unstimulated (grey/filled triangles) or stimulated (grey/empty triangles), and low DO treatment groups, either unstimulated (red/filled circles) or stimulated (red/empty circles) after one, two and three days post intraperitoneal stimulation with inactivated *Aeromonas hydrophila* cells. Genes involved in stress (green) or immune response (blue) are either lowly (**A**), moderately (**B**) or highly (**C**) expressed. Data points represent mean of four individual samples (+SEM), calculated per 100 ng of total RNA. Statistically significant differences were calculated via one-way ANOVA followed by Tukey‘s multiple comparison test (*p* = 0.05).

**Figure 9:**
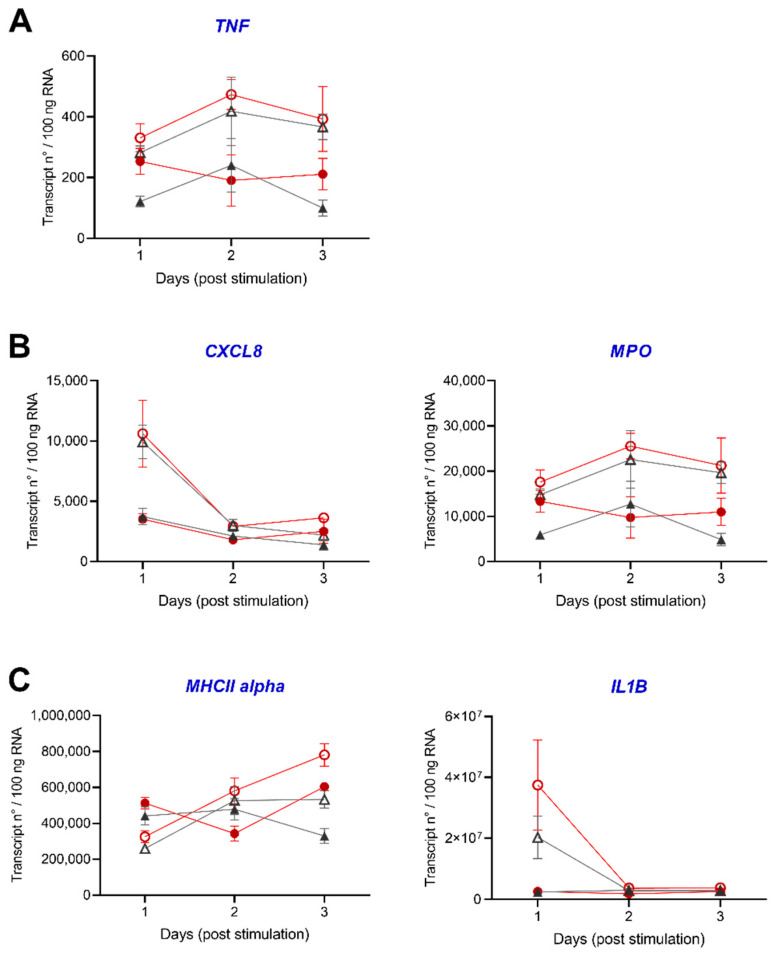
Expression of stress and immune markers in spleen of pikeperch upon acute peritoneal inflammation. Relative expression of candidate genes in spleen of pikeperch. Normoxic control group (grey), either unstimulated (grey/filled triangles) or stimulated (grey/empty triangles), and low DO groups, either unstimulated (red/filled circles) or stimulated (red/empty circles) after one, two and three days post intraperitoneal stimulation with inactivated *Aeromonas hydrophila* cells. Genes involved in stress (green) or immune response (blue) are either lowly (**A**), moderately (**B**) or highly (**C**) expressed. Data points represent mean of four individual samples (+SEM), calculated per 100 ng of total RNA. Statistically significant differences were calculated via one-way ANOVA followed by Tukey‘s multiple comparison test (*p* = 0.05).

We further aimed to evaluate the transcriptomic changes orchestrating the acute inflammation using established rt-qPCR analysis (Figure 8A–C and Figure 9A–C). Ten genes (*CSF2*, *EPAS1*, *FTH1*, *HIF1A*, *HMOX1*, *HSF1*, *HSF2*, *HSP90AA1*, *NR3C1* and *RAG1*) were exclusively determined in HK with additional five genes (*CXCL8*, *IL1B*, *MHC II alpha, MPO* and *TNF*) in both tissues. *TNF* was exclusively detectable in the spleen, with numbers only below 10 transcripts per 100 ng RNA in HK (data not shown).

We detected the lowest copy numbers with transcripts only below 4 × 10^3^ transcripts/100 ng RNA for five genes (*CXCL8*, *TNF*, *HMOX1*, *HSP90AA1* and *RAG1*) in HK and spleen of the individual fish (Figure 8A and Figure 9A). For the three genes *HSF1*, *IL1B* and *MHCII alpha*, we determined copy numbers above 5 × 10^5^ per 100 ng RNA in HK and spleen (Figure 8C and Figure 9C).

In HK, we observed highly dynamic expression profiles of the selected genes. An intraperitoneal stimulation decreased the transcript levels of *FTH1*, *HMOX1*, *HIF1A* and *HSP90AA1* after two days of the experiment in the control and low DO group. *EPAS1*, *NR3C1* and *RAG1* showed lower copy numbers in stimulated control and low DO fish than the unstimulated control group on any day of the experiment. *CXCL8* transcript numbers decreased after stimulation in the control group and increased in the low DO group after two days. The transcript numbers of *MHCII alpha* increased two days post-stimulation in both groups (control and low DO).

In the spleen, the intraperitoneal stimulation led to a remarkable increase in expression of the genes coding for the inflammatory cytokines CXCL8 and IL1B, which reduced in comparison to the levels seen in unstimulated fish two days post-stimulation. In contrast, *MHCII alpha*, *MPO* and *TNF* transcript levels increased in both groups (control and low DO) two days post-stimulation.

## 4. Discussion

### 4.1. The Hypoxic Challenge of 40% DO Does Not Induce Substantial Changes in Major Health Parameters

Exposure to stress stimuli initiates a cascade of physiological mechanisms, allowing the mobilization of energy to cope with stressors and restore homeostasis. In teleost fish, like in other vertebrates, several markers, including blood parameters and health indices, are frequently applied as surrogates of the adaptation response. Apart from the spleno-somatic index, reflecting the immune status, or hepato-somatic index, representing the organism’s metabolic rate, the blood cortisol serves as the primary indicator of the ongoing stress response. Its increasing levels regulate a vast array of processes directing the energy metabolism toward the mobilization of hepatic glycogen and an increased availability of glucose to facilitate the successful adaptation to the stressor [32,33,34,35]. Unexpectedly, none of the examined parameters showed levels outside the physiological range or significant differences between the low DO group and the control group in the current study. Cortisol is an excellent marker for acute stress, including low DO levels, but its reliability under chronic conditions is uncertain due to its fast release and clearance [36,37,38,39].

The levels of free glucose are in line with former studies in pikeperch, whereas observed lactate levels were lower [40,41]. Both physiological parameters have been shown to increase after an acute short-term low DO event [42]. However, our findings are concordant with the study of O’Connor et al. (2011), in which different populations of three-spined stickleback (*Gasterosteus aculeatus*) showed no significant changes in whole-body cortisol glucose and lactate levels after a week of low oxygen conditions (2.2 mg/L DO) [43]. Similarly, Douxfils et al. (2014) reported a fast return to basal cortisol and glucose levels after a response to low oxygen saturation in juvenile Eurasian perch [44]. In common carp (*Cyprinus carpio*), unaltered HSI was detected after long-lasting low DO levels [45]. Overall, the absence of significant changes in the studied health parameters indicate relatively high tolerance of pikeperch to low levels of DO and high pace of adaptation responses preserving the homeostasis even at ±3.2 mg/L DO.

### 4.2. Effects of Low DO on Cell Distribution and Gene Expression

Previous studies have shown that inadequately low oxygen saturation results in modifications of the innate and adaptive immunity in fish and alters the cell composition in main lymphoid organs [46,47,48,49,50,51,52]. In the presented study, we employed flow cytometry to elucidate the changes induced by the chronic exposure to low DO in blood and head kidney. Interestingly, our results revealed an increased proportion of myeloid cells, both in circulation and in the lymphoid organs of low DO fish, reflecting a potentially higher mobilization of the immune system. This observation belongs to one of the hallmarks of the conserved transcriptional response to adversity and is in accord with an increased rate of circulating myeloid cells in the blood of maraena whitefish (*Coregonus maraena*) exposed to crowding stress [53], higher mobilization of myeloid cells to the blood of gilthead seabream (*Sparus aurata*) after exposure to short-term stress [54] or increased number of circulating myeloid cells observed in mammalian models of stress responses [55,56]. Induction of erythropoiesis at low DO levels, which increases oxygen transport in the blood, has been observed previously in teleosts [57]. Nevertheless, we did not observe any substantial increase in the number of circulating erythrocytes throughout the experiment (data not shown). Therefore, oxygen levels of ±3.2 mg/L DO may be classified as a hypoxic condition for pikeperch, but not as a situation of severe hypoxia.

The detected transcript patterns for the eight examined stress- and immune-relevant genes further illustrate the weak response to low DO. Although the examined genes were previously demonstrated to be responsive to hypoxic conditions or belong to downstream targets of hypoxia-inducible factor 1 alpha (HIF1A), which regulates the hypoxia response pathway, our analysis revealed only slight downregulation of their expression. More specifically, HIF1A is essential for the response of hypoxic fish with complex physiological and biochemical modifications involving the immune system [58,59]. In large yellow croaker (*Larimichthys crocea*), severe hypoxic conditions (1.6 ± 0.2 mg/L DO) for two days resulted in an upregulation of most immune genes, as well as *HIF1A*, in HK [60]. According to these investigations, we expected significant changes within the transcription in hypoxic challenged pikeperch, with increased expression of *HIF1A* and other stress marker genes such as *NR3C1* and *HSP90AA1*. However, we detected a prominent down-regulation of *HIF1A* transcript numbers in the head kidney of pikeperch, with significant differences at days 14 to 28 of the experiment, but relatively stable transcript levels in the liver. In European bass (*Dicentrarchus labrax*), acute hypoxic conditions of 1.9 mg/L DO for 4 h, and chronic conditions of 4.3 mg/L DO for 15 days cause an elevated *HIF1A* transcription in the liver [61]. The hypoxia-sensitive percid species Eurasian perch showed up-regulated HIF1A transcription in the liver after an acute hypoxic oxygen saturation of 0.4 ± 0.1 mg/L DO for 1 h, but not after 15 days of 2.8 ± 0.3 mg/L DO [62]. Mohindra et al. (2013) observed in the hypoxia-tolerant Indian catfish (*Clarias batrachus*) a significantly up-regulated expression of HIF1A in the liver and down-regulation in the head kidney after 1 h of 0.98 mg DO per liter. Whereas, after another 5 h HIF1A was significantly up-regulated in the head kidney [63]. A down-regulation of HIF1A transcription in response to hypoxia stress was suggested to be the outcome of a hypoxia shock [64,65]. They concluded that the transcriptional regulation of HIF1A is a complex and tissue- and species-dependent process. This further suggest that the range of tolerance of pikeperch reared in intensive aquaculture is hardly impacted at DO saturations of 40%. It did not establish a severe stress response or severe immune suppression within 28 days. Collected data rather indicate an ongoing adaptation process already after 24 h lasting till day 21. Nevertheless, the obtained gene expression data are based on preselected candidate genes and global gene expression analyses, such as RNAseq or microarray-based analyses, might uncover yet not considered but regulated genes and pathways after DO decrease that influence or could affect homeostasis and fish welfare.

### 4.3. Acute Inflammation Is Moderately Influenced by Low DO

Previously, several observations suggested a negative impact of hypoxic conditions on the fish immune system [48,66,67]. To provide a deeper understanding of this phenomenon, we used a previously established model of acute peritoneal inflammation to evaluate how, and to what extent, acute inflammation is impaired by low DO. Generally, the processes driving the acute inflammation followed a pattern described previously in other fish species [28,68]. The injection of inactivated *A. hydrophila* induced a rapid mobilization of myeloid cells from head kidney and their release into the circulation [69,70]. Consequently, within 24 h post-injection, we observed increased SSI in both stimulated groups and a considerably increased expression of the myeloperoxidase (MPO), a key marker of granulocytes in the spleen [50]. In spleen, the detected increase of MPO lasted till the end of the experiment at 72 h. In the head kidney, a stimulation resulted in reduced transcript levels of MPO in both groups reflecting the efflux of granulocytes into the circulation. A depletion of neutrophils in head kidney after peritoneal inflammation has been detected in the goldfish (*Crassius auratus*) by Bielek et al. (1999) [71]. Simultaneously, we observed a dramatic increase in the number of myeloid cells in the peritoneal niche. Notably, in line with the aforementioned results, only marginal differences in the number of recruited cells were seen between the normal and low DO fish. On a molecular level, the rapid recruitment of myeloid cells into the peritoneal cavity was complemented by the increased production of proinflammatory cytokines (particularly CXCL8 and IL1B) in the spleen and head kidney of studied fish [72,73]. For both cytokines, the increase in gene expression was more pronounced in the stimulated spleen. Within HK the production of the major pro-inflammatory cytokine TNF was independent of the treatment marginally low (data not shown) [74]. While in spleen, higher expression was detectable for both groups one day post-injection and this increase persisted until the third day of the stimulation. The primary source of TNF is activated macrophages which, therefore, might also be involved in the detectable increased SSI [72].

Within the following 24 h, we witnessed a resolution of the acute inflammation, manifested by decreasing expression of inflammatory cytokines, reduced presence of myeloid cells in the circulation and an influx of lymphocytes into the peritoneal cavity. Strikingly, although we did not observe a strong influence of hypoxia on the recruitment of myeloid cells, the number of lymphocytes differed considerably between both groups, reaching almost three times lower numbers in the low DO group.

Taken together, these findings support the notion of dichotomy in the effect of hypoxia on the innate and adaptive arm of immunity, suggested by previous findings from mammalian models. Thus, while the innate immune cells, and granulocytes in particular, are better equipped to maintain viability and functionality under hypoxic conditions, the lymphocytes require high energy metabolism coupled with sufficient oxygen availability for their survival and effective development of effector functions [75,76]. Similarly, in the present study, the recruitment, and the effector functions of the myeloid during the acute inflammation were comparable between both groups, while the influx of lymphocytes was impaired by the low DO, suggesting high evolutionary conservation of these processes in the tree of life.

## 5. Conclusions

In their natural habitats, fishes would avoid low oxygen levels by simply escaping the current situation. However, this option is not available in rearing tanks of intensive aquaculture facilities. In the present study, we evaluated the effect of hypoxic conditions (±3.2 mg/L DO) on the health and immune status of pikeperch reared in RAS. We defined stable blood parameters, slightly downregulated gene expression (*FTH1*, *HIF1A* and *NR3C1*) and a functional acute inflammatory response towards bacterial stimulation. Our results confirmed that pikeperch do not develop severe responses or immunosuppression at hypoxic conditions and together with our previous study investigating the challenge of rising water temperatures in pikeperch [29], indicates that pikeperch in aquaculture may not be as sensitive to common environmental stressors as previously thought.

## Figures and Tables

**Table 1:** Gene-specific primers used in this study.

Gene Symbol	Official Names	Sense Primer (5′-3′)	Antisense Primer (5′-3′)	Primer Efficiency [%]	Fragment Length [bp]
Reference genes:					
EEF1A1	Elongation factor 1 alpha	ATGGACAGACCCGTGAGCATG	TTCTTGATGTAGGTGCTCACTTC	105	151
RPL32	Ribosomal protein L32	GGCGTAAACCCAGAGGTATTGA	ACCTCGAGCTCCTTGACATTGT	105	157
RPS5	Ribosomal protein S5	GCAGGATTACATTGCTGTGAAAG	TCATCAGCTTCTTGCCATTGTTG	101	161
Target genes:					
Stress response					
EPAS1	Endothelial PAS domain protein1	AGTGCAGAGGACGCACAGATG	TCATGTTCACCTGCGTGAGCC	100	139
HIF1A	Hypoxia inducible factor 1 subunit alpha	CCAGTCGAATCCCTTGAGAGTT	CTGTGGGGTCCTCTTAGCAAC	97	156
HMOX1	Heme oxygenase 1	GCTCGCTGTATGAGGTCTACC	TCTCTCCAGTCCTGGCCATAG	101	154
HSP90AA1	Heat shock protein 90 alpha family class A member 1	AGATACTACACCTCGGCTTCTG	TCACCAGTGATGTAGTAGATGTG	100	101
HSF1	Heat shock transcription factor 1	TGTGTCTTGTGCAGAGTGGAAC	GCTGGCCATGTTGTTGTGTTTG	111	101
HSF2	Heat shock transcription factor 2	AGCCGTCCCGCAGCTCCCT	CGGGACTCAGTTCGCACAGG	91	93
NR3C1	Nuclear receptor subfamily 3 group c member 1	CCAGTCCTGCATGGATTCACTT	AGGTCCATAGTGTTGTCACTGAA	100	180
Immune response					
CSF2 *	Colony stimulating factor 2	CCAGCAGGAATACACAGAAATCT	CGCAGATAGAGACAATGATGAAG	95	164
CXCL8 *	Interleukin 8	AACAGGGATGAGTCTGAGAAGC	GCTTGGAAATGAAGTCTTACATGA	98	158
FTH1	Ferritin heavy chain 1	AGAACTGGCAGACTGGGTGAC	CTGCTTTCTTTGCCCAGGGTG	99	102
IL1B *	Interleukin 1 beta	TCGACCTACTTGCACCCTACA	TCTGCCTCCACAACCTGAA	101	137
MHC II alpha *	Major histocompatibility complex II alpha	TGGACCAACCACTGACCAGAAT	CATCATCAGTCCCAGCCAATCA	99	168
MPO *	Myeloperoxidase	GTTTGATCGGCCGTCCTGCTA	ATTACCAGCCAAGCCATGGTCA	98	152
RAG1 *	Recombination activating 1	CTCAGGCTTCAGTGTCATGATC	AACCTCTTTCTCCTCCTCGTCT	95	157
TNF *	Tumor necrosis factor	GTCTTTGGAACCAGGCTATTTAC	TTTATGCCTCAGGCTTGACTGG	89	157

* Genes applied exclusively for stimulation experiment.

## Data Availability

The data presented in this study are available in this manuscript and its supplementary materials and are available on request from the corresponding authors.

## Supplementary Materials

The following are available online at https://www.mdpi.com/article/10.3390/biology10070649/s1, Table S1: Individual values of blood and health parameters measured in this study.
