# Supplementary material for: Effects of Chronic Hypoxia on the Immune Status of Pikeperch (Sander lucioperca Linnaeus, 1758)
Source: Biology (Basel). 2021 Jul 12;10(7):649. doi: 10.3390/biology10070649 (PMC8301350; doi:10.3390/biology10070649)
Supplement: Supplementary file 1 [file biology-10-00649-s001.zip › biology-1263773-supplementary.pdf]

Table S1. Individual values of blood and health parameters measured in this study.

A		Day1 Mean ± S.E.M	Day7 Mean ± S.E.M	Day14 Mean ± S.E.M	Day21 Mean ± S.E.M	Day28 Mean ± S.E.M
Cotrisol (nmol/L)	Control	n.a.	n.a.	n.a.	n.a.	n.a.
	Treatment	n.a.	n.a.	n.a.	n.a.	n.a.
Glucose (mmol/L)	Control	5.80 ± 1.55	4.76 ± 0.85	3.72 ± 0.99	4.74 ± 0.81	6.01 ± 1.92
	Treatment	5.68 ± 0.48	6.07 ± 0.77	5.91 ± 1.00	3.12 ± 0.21	4.22 ± 1.11
Lactate (mmol/L)	Control	2.62 ± 0.68	0.68 ± 0.42	0.65 ± 0.22	0.60 ± 0.25	1.70 ± 0.44
	Treatment	3.08 ± 2.89	0.76 ± 0.37	0.92 ± 0.41	0.00 ± 0.00	1.34 ± 0.22
HSI	Control	1.40 ± 0.11	1.43 ± 0.11	1.29 ± 0.07	1.48 ± 0.13	1.38 ± 0.09
	Treatment	1.23 ± 0.11	1.39 ± 0.04	1.31 ± 0.08	1.27 ± 0.07	1.16 ± 0.06
SSI	Control	0.04 ± 0.00	0.03 ± 0.01	0.05 ± 0.00	0.04 ± 0.00	0.05 ± 0.00
	Treatment	0.03 ± 0.00	0.12 ± 0.59	0.04 ± 0.00	0.03 ± 0.00	0.04 ± 0.00
B		Day 1 p.s.	Day 2 p.s.	Day 3 p.s.		
SSI	Control unstimulated	0.05 ± 0.00	0.06 ± 0.01	0.06 ± 0.01		
	Treatment unstimulated	0.04 ± 0.01	0.05 ± 0.00	0.05 ± 0.00		
	Control stimulated	0.08 ± 0.01	0.07 ± 0.00	0.07 ± 0.00		
	Treatment stimulated	0.08 ± 0.01	0.06 ± 0.00	0.06 ± 0.01		

HSI = hepato-somatic index, p.s. = post stimulation; S.E.M. =standard error of the mean; SSI = spleen somatic index; A: n = 5 fish per group and day, B: n = 4 fish per group and day.
